# Role of 11C Methionine Positron Emission Tomography (11CMETPET) for Surgery and Radiation Therapy Planning in Newly Diagnosed Glioblastoma Patients Enrolled into a Phase II Clinical Study

**DOI:** 10.3390/jcm10112313

**Published:** 2021-05-25

**Authors:** Federico Pessina, Pierina Navarria, Elena Clerici, Luisa Bellu, Andrea Franzini, Davide Milani, Matteo Simonelli, Pasquale Persico, Letterio S. Politi, Alessandra Casarotti, Bethania Fernandes, Simone Olei, Martina Sollini, Arturo Chiti, Marta Scorsetti

**Affiliations:** 1 Neurosurgery Department, IRCCS Humanitas Research Hospital, Rozzano, 20089 Milan, Italy; federico.pessina@hunimed.eu (F.P.); andrea.franzini@humanitas.it (A.F.); davide.milani@humanitas.it (D.M.); alessandra.casarotti@humanitas.it (A.C.); simone.olei@humanitas.it (S.O.); marta.scorsetti@hunimed.eu (M.S.); 2 Department of Biomedical Sciences, Humanitas University, Pieve Emanuele, 20090 Milan, Italy; matteo.simonelli@hunimed.eu (M.S.); Letterio.politi@hunimed.eu (L.S.P.); arturo.chiti@hunimed.eu (A.C.); 3 Radiotherapy and Radiosurgery Department, IRCCS Humanitas Research Hospital, Rozzano, 20089 Milan, Italy; Elena.Clerici@humanitas.it (E.C.); luisa.bellu@humanitas.it (L.B.); 4 Oncology and Hematology Department, IRCCS Humanitas Research Hospital, Rozzano, 20089 Milan, Italy; pasquale.persico@humanitas.it; 5 Neuroradiology Department, IRCCS Humanitas Research Hospital, Rozzano, 20089 Milan, Italy; 6 Pathology Department, IRCCS Humanitas Research Hospital, Rozzano, 20089 Milan, Italy; bethania.fernandes@humanitas.it; 7 Diagnostic Imaging Department, IRCCS Humanitas Research Hospital, Rozzano, 20089 Milan, Italy

**Keywords:** newly diagnosed glioblastoma, [11C]-methionine PET, surgery, radiation therapy, supratotal resection

## Abstract

(1) Background: We investigated the role of [11C]-methionine PET in a cohort of newly diagnosed glioblastoma multiforme (GBM) patients to evaluate whether it could modify the extent of surgical resection and improve radiation therapy volume delineation. (2) Methods: Newly diagnosed GBM patients, ages 18–70, with a Karnofsky performance scale (KPS) ≥ 70 with available MRI and [11C]-methionine PET were included. Patients were treated with different amounts of surgical resection followed by radio-chemotherapy. The role of [11C]-methionine PET in surgical and RT planning was analyzed. A threshold of SUVmax was searched. (3) Results: From August 2013 to April 2016, 93 patients were treated and included in this analysis. Residual tumor volume was detected in 63 cases on MRI and in 78 on [11C]-methionine PET, including 15 receiving gross total resection. The location of uptake was mainly observed in FLAIR abnormalities. [11C]-methionine uptake changed RT volume in 11% of patients. The presence of [11C]-methionine uptake in patients receiving GTR proved to influence survival (*p* = 0.029). The threshold of the SUVmax conditioning outcome was five. (4) Conclusions: [11C]-methionine PET allowed to detect areas at higher risk of recurrence located in FLAIR abnormalities in patients affected by GBM. A challenging issue is represented by integrating morphological and functional imaging to better define the extent of surgical resection to perform.

## 1. Introduction

The current standard of care for newly diagnosed glioblastoma (GBM) is maximal safe resection, followed by adjuvant radiation therapy (RT) with concurrent and adjuvant temozolomide chemotherapy (TMZCHT) [1,2]. Notwithstanding, gains have been obtained but the results are still unsatisfactory, and almost all cases relapse in the site of primary treatment. A way forward in improving the outcome might be more precise identification of the volume at the most significant risk of recurrence. The critical point is reaching correct tumor delineation based on imaging, aiming to understand the actual degree of normal brain infiltration, and optimizing the extent of surgical resection. The contrast enhancement T1-weighted-MRI usually outlines the most aggressive tumor area but leads to underestimating the tumor-infiltrating border. Fluid-attenuated-inversion-recovery (FLAIR) is a T2-weighted MR sequence with suppression of the cerebral spinal fluid (CSF) signal. FLAIR provides a better delineation of the lesion once the confounding effect of CSF is removed. Therefore, this sequence allows a more accurate definition of the infiltrating microscopic disease growing outside the enhanced area on T1 MRI [3,4]. Recent international guidelines suggest relying on FLAIR–MRI imaging modality in tumor burden delineation [5]. More recently, different reports suggest that the use of carbon-11-methionine positron emission tomography ([11C]-methionine PET ([11C]MET PET) improves diagnostic accuracy for both diagnosis and treatment planning of brain tumors [6,7,8,9,10,11,12,13,14,15,16]. A high uptake by the glioma cells compared to the healthy brain tissue characterizes this tracer. While MRI gives a morphological tumor delineation, [11C]MET PET provides complementary metabolic information, allowing more precise viable tumor cell identification. Evidence regarding the predictive and prognostic value is still lacking, and its use in planning local treatments, such as surgery and radiation therapy, is debated. In 2013, we designed a Phase II trial to evaluate the role of a short RT course in newly diagnosed GBM patients, and results are already published [17]. All patients underwent a hypofractionated RT (HFRT) scheme following different entities of surgical resection. The extent of surgical resection (EOR) was defined based on MRI, while [11C]MET PET along with MRI was employed to identify residual biological tumor volume (BTV). In this second analysis, we investigated the role of [11C]MET PET in this cohort of newly diagnosed GBM patients enrolled in a phase II clinical study. The aims were to evaluate whether the employment of metabolic imaging ([11C]MET PET) along with morphological ones (MRI), could provide additional information to maximize the EOR and/or to improve the target volume delineation for RT planning.

## 2. Materials and Methods

### 2.1. Patients

Newly diagnosed GBM patients, ages 18–70 years, with a Karnofsky performance scale (KPS) ≥ 70, a residual tumor or surgical cavity with a maximum diameter of 10 cm, normal liver, kidney and bone marrow functions with available MRI and MET PET, were included in the present analysis. All patients provided written informed consent to the treatment and the use of their data for scientific purposes. The trial was registered at the ClinicalTrials.gov site with the number NCT00006353.

### 2.2. Surgery

Surgery was performed in all patients to remove tumors according to functional boundaries. Tumor removal was achieved with the aid of brain-mapping techniques and imaging neuro-navigation (post-contrast T1 weighted images, FLAIR, functional MRI, DTI) coupled with intraoperative ultrasounds to afford maximal resection and maintenance of full patient functional integrity. The extent of resection (EOR) was determined by comparing preoperative post-contrast T1 weighted MRI with postoperative MRI study, acquired within 48 h after surgery, and were calculated as follows: preoperative tumor volume—postoperative tumor volume/preoperative tumor volume %. Gross total resection (GTR) was defined as amount of surgical resection 95–100%, subtotal resection (STR) 78–94%, partial resection (PR) 30–77%, and biopsy < 30%. The contrast-enhanced residual tumor volume (CERTV) was also identified on postoperative MRI. The tumor molecular profile was available in all cases. Immunohistochemical staining for isocitrate dehydrogenase (IDH1/2) was performed on BenchMark XT automated tissue staining systems (Ventana Medical Systems, Inc., Tucson, AZ, USA), using validated protocols. O-6-methylguanine-DNA methyltransferase (MGMT) promoter methylation status was determined by pyrosequencing (Diatech Pharmacogenetics, Jesi, Ancona, Italy; MGMT plus, valid CE/IVD).

### 2.3. Radiation Therapy

For the radiation therapy planning CT scan, T1-weighted FLAIR (fluid-attenuated inversion recovery images) and T2-weighted 3D-FLAIR followed by T1-weighted MPRAGE MRI and [11C]MET PET were acquired within 1 month from surgery. Images were co-registered with each other. A total amount of 300–500 MBq carrier-free L-(methyl-11C) methionine was administered to patients who had been fasting for at least four hours and images were acquired 10–15 min later. CT attenuation-corrected 3D images were acquired for 10 min, and the images were subsequently reconstructed using an iterative reconstruction algorithm (OSEM) and displayed on GE Xeleris Workstation. The definition of the pathologic uptake was determined based on visual assessment and semi-quantitative evaluation with a standardized uptake value (SUVmax) by applying a reference tumor-to-background (TBR) ratio of 1.5. We used healthy appearing reference brain tissue uptake values as the background, calculated within a crescent-shaped volume of interest in the contralateral parenchyma. The maximum TBR (TBRmax) was calculated by dividing the SUVmax of the lesion by the mean SUV of healthy appearing brain parenchyma [18,19]. 

The biological tumor volume (BTV) was defined as the volume and the uptake area (SUVmax) on the [11C]MET PET. We dichotomized the SUV max in increments of 0.1 to eventually obtain the maximum significance in the difference between the two groups’ outcomes. Two different clinical target volume (CTV) were outlined: CTV1 corresponded to the entire surgical cavity plus the residual tumor after surgery or the abnormality on the T1-weighted post-contrast MPRAGE and MET PET in the case of biopsy; in cases of uptake on 11CMETPET outside of the surgical cavity or residual tumor, it was included in CTV1. CTV2 corresponded to the abnormality on FLAIR MRI images after surgery and included, in all cases, CTV1 and 11CMETPET uptake.

Planning target volumes 1 and 2 (PTV1/PTV2) were generated, adding an isotropic margin of 5 mm from CTV1 and CTV2, respectively. Intensity-modulated radiation therapy was performed within 4–6 weeks after surgery, using volumetric modulated arc therapy (VMAT). The dose prescribed was 60 Gy with a daily fraction of 4 Gy on PTV1, and 42 Gy with a daily fraction of 2.8 Gy on PTV2 for 15 consecutive days, using a simultaneous integrated boost (SIB). Organs at risk (OARs) outlined were optic nerves and chiasm, lens, brainstem and cochlea without additional margins, and the recommended maximal doses were ≤ 40 Gy, ≤ 10 Gy, ≤ 30 Gy, and ≤ 30 Gy, respectively. The dose was prescribed to an isodose line that ensured that more than 98% of PTV1-2 received 95% of the prescribed dose. In each session, a patient position check was performed, using the ExacTrac (Brainlab, Feldkirchen, Germany) system and cone-beam computer tomography (CBCT).

Correlation between postoperative CERTV and BTV was performed. Additionally, modifications of CTV1 and CTV2 due to the MET PET uptake were recorded.

### 2.4. Chemotherapy

All patients received TMZ concurrently with HFRT. TMZ was administered orally, once daily, at 75 mg/m^2^, starting on the first day of HFRT and continuing for the whole treatment. After a 4 week break, adjuvant TMZ was administered at 150 to 200 mg/m^2^ orally, once daily, for five consecutive days every 28 days up to 12 cycles, or until disease progression occurred.

### 2.5. Supportive Care

Corticosteroids were administered during the whole HFRT treatment and were progressively reduced at the end of RT. Antiepileptic drugs (AEDs) were prescribed only in patients with a history of at least one seizure. The most frequently used AEDs were levetiracetam as a first-line instance followed by topiramate, lamotrigine or lacosamide.

### 2.6. Outcome Evaluation

The clinical outcome was evaluated by neurological examination and MRI imaging one month after concurrent CHT–HFRT and every four months after that. The MET PET was performed at 4 and 12 months during maintenance CHT or to rule out pseudo-progression. According to the Response Assessment in Neuro-Oncology (RANO) working group [20], tumor progression was defined. Thirty-days postoperative morbidity and mortality were registered. The appearance of new neurological deficits lasting for more than 30 days after surgery, or the worsening of preoperative ones, were defined as major complications. All other complications were defined as minor. Neuropsychological assessment was performed through a shortened version of the “Milano Bicocca Battery”, evaluating language, memory, apraxia, visuo–constructional abilities, and attentive and executive functions [21]. Patients were evaluated one week before the start of HFRT and 1, 6, and 12 months thereafter.

### 2.7. Statistical Analysis

The original study design was previously published [17]. In the present analysis, we evaluated MET PET’s role in eventually integrating the EOR definition by MRI, the correlation between CERTV and BTV, and the variation of RT target volume delineation concerning the BTV. A threshold of SUVmax was searched, using 0.1 increments. The influence of these factors in the outcome prediction was evaluated. Survival and recurrence time observations were evaluated according to the Kaplan–Meier method, starting from the date of diagnosis. The median survival time was evaluated, obtaining from Ŝ(t) the Kaplan–Meier product-limit estimate of the survivor function. Confidence bounds of the survivor function were calculated based on the asymptotic variance of ln[−ln Ŝ(t), as described in Kalbfleisch and Prentice. The upper (lower) confidence limits for the median survival times are defined as the first time at which the upper (lower) confidence limit for Ŝ(t) is less than or equal to 0.5. A not reached indicator (nr) was specified if the survival estimate resulted above the 50% level in the considered observation time. The upper confidence bound of median survival time was labeled as one if not evaluable with the above method for a specific group of patients in the considered time of observation. The log–rank test was used to assess the prognostic role of the different individual variables for dichotomous variables gender, MGMT, IDH, presence of CERTV, BTV, SUVmax, and TBRmax. The univariate Cox model was applied for the remaining variables. A multivariate Cox regression model was used to estimate our variable’s independent association with overall survival [22]. A statistical analysis was performed using the Medical software, version 17.7 (MedCalc Software, Ostend, Belgium).

## 3. Results

From August 2013 to April 2016, out of 97 newly diagnosed GBM patients enrolled on trial, 93 had MRI and MET PET imaging available and were included in the present analysis. The median age was 61 years (range 23–77 years), and a large part of them had KPS 90–100. Surgery followed by HFRT was performed in all 93 patients. Concurrent and adjuvant TMZ was performed in 91 (97.8%) patients and omitted in 2 (2.2%) for liver disorders or hematologic toxicity. Patients, tumor, and treatments characteristics are shown in Table 1.

Regarding the amount of surgical resection, the GTR of the enhanced tumor was obtained in 45 (48.3%) patients, STR in 18 (19.4%) patients, and PR in 16 (17.2%). CERTV detected on post-contrast postoperative MRI was observed in 63 (67.7%) patients, with a median volume of 4.23 cm^3^ (range 0.17–35.33 cm^3^). The BTV was recorded on [11C]MET PET in 78 (83.9%) cases and the median BTV was 8.47 cm^3^ (0.11–62.20 cm^3^). In relation to the EOR, in all 48 patients who received biopsy, subtotal or partial resection, the BTV was located in the residual tumor. In patients who underwent GTR, the BTV was placed both along the margin of the surgical cavity, and in FLAIR abnormalities in 15/45 patients (33.3%). An uptake outside the surgical cavity into the FLAIR abnormalities region without a CERTV was observed in 15 (33.3%) patients, up to a distance of 30 mm (range 11–30 mm) (Table 2 and Table 3).

**Figure 1:**
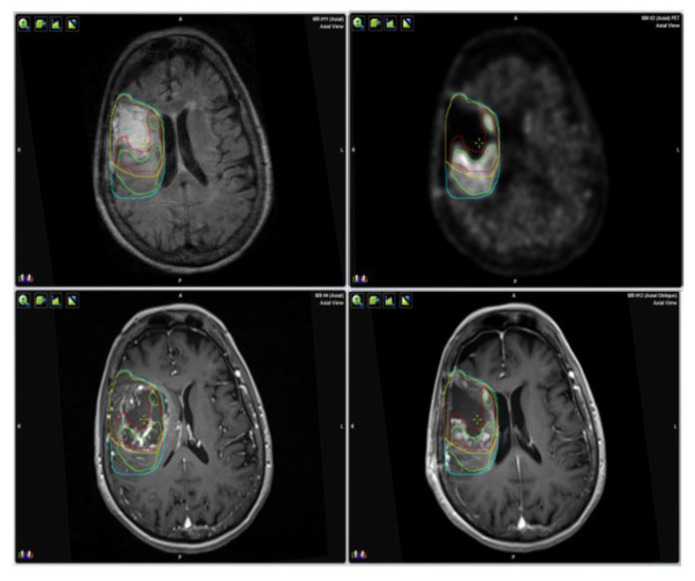
Volumes definition on post-contrast MRI, FLAIR, MRI and 11CMETPET. Clinical target volume 1 (CTV1)—yellow line; clinical target volume 2 (CTV2)—light blue; contrast-enhanced residual tumor volume (CERTV)—red; 11METPET uptake—green.

Concerning HFRT, the median CTV1, corresponding to the entire surgical cavity plus the contrast enhancing residual tumor after surgery, was 76.65 cm^3^ (range 23.98–190 cm^3^); the median CTV2, corresponding to the abnormality on FLAIR MRI images after surgery was 119.5 cm^3^ (range 23.98–321 cm^3^). In all cases, the CTV1 was fully included in the CTV2. In all cases, the whole BTV was inside the CTV1, while in 10 (11%) patients, part of the BTV was outside the CTV1 in the FLAIR abnormalities area, and the CTV1 was modified in relation to the 11CMETPET uptake. An example of CERTV, BTV, CTV1 and CTV2 volume definition is shown in Figure 1.

### 3.1. Progression-Free Survival (PFS) and Overall Survival (OS) Analysis

**Figure 2:**
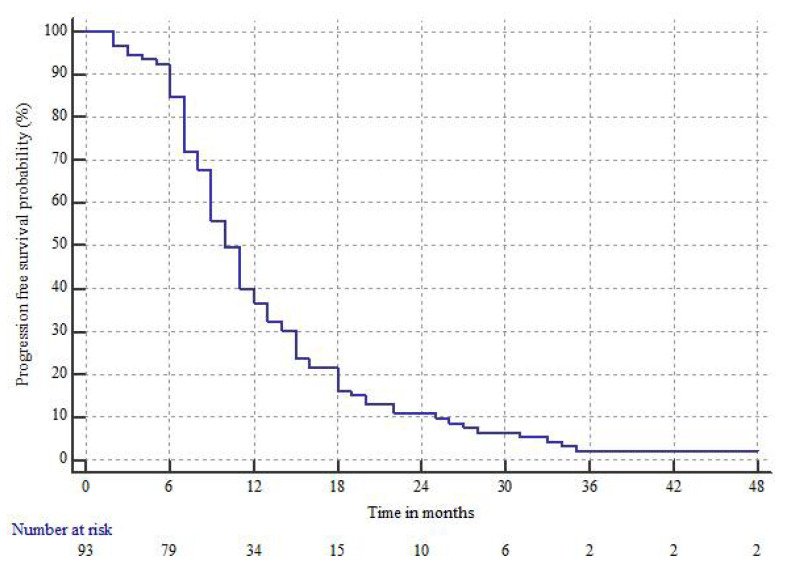
Progression free survival (PFS).

The median follow-up time was 72 months (range 58–90 months). Recurrences occurred in 91 (97.8%) patients; it was local in 74 (81.3%), local and distant in 13 (14.3%) and only distant in 4 (4.4%). The median PFS time and the 1, 2, 3 year PFS rates were 10 months (95%CI 9–11), 36.6 ± 4.9%, 10.8 ± 3.2%, and 2.1 ± 1.5%, respectively, as shown in Figure 2.

**Figure 3:**
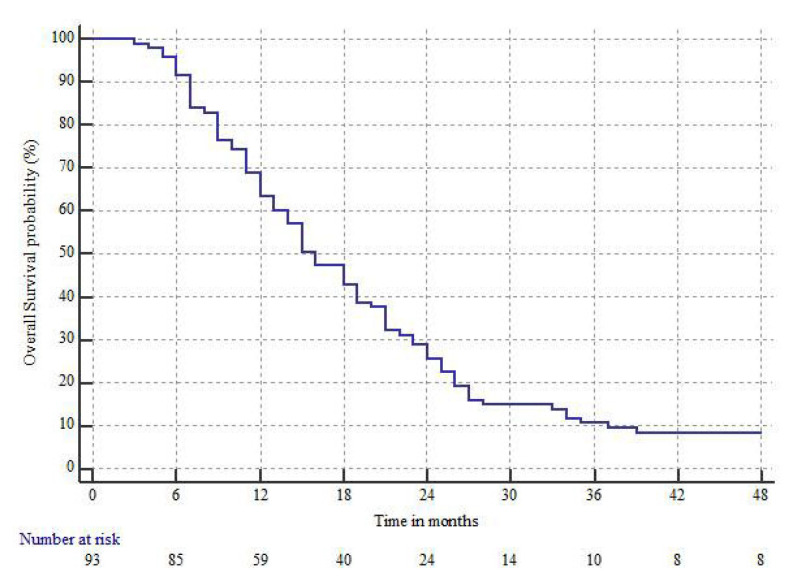
Overall survival (OS).

At the final observation time, 91 patients were dead and 2 alive. The median OS time and the 1, 2, 3 year OS rates were 16 months (95%CI 14–19), 63.4 ± 4.9%, 25.8 ± 4.5%, and 10.8 ± 3.2%, respectively, as shown in Figure 3.

### 3.2. Prognostic Factors Analysis

Outcomes according to prognostic factors, including age, MGMT status, evidence of CERTV and BTV, the correlation between BTV and CERTV, SUVmax, and TBRmax were analyzed. The highest benefit was observed in treated patients with age ≤ 60 years and MGMT methylated tumor, having undergone GTR resection without any evidence of CERTV and BTV. The threshold of SUVmax conditioning survival was 5 (range 4.7–5.1). Patients with a TBRmax higher than 4.5 showed a significant worse prognosis than those with TBRmax ≤ 4.5 with a negative trend from 1 to 3 year survival.

**Figure 4:**
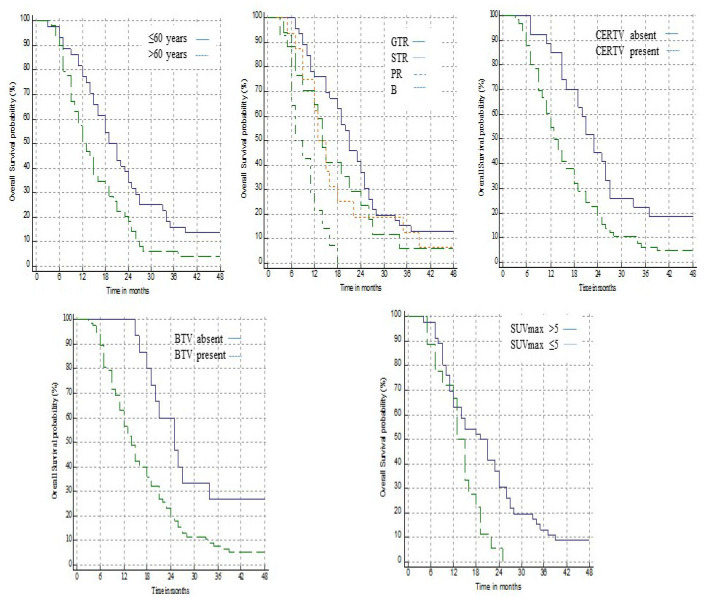
Prognostic factors impacting overall survival. GTR—gross total resection; STR—subtotal resection; PR—partial resection; CERTV—contrast enhances residual tumor volume; BTV—biological tumor volume.

Regarding PFS, a favorable trend was recorded in cases of younger patients with methylated MGMT and without residual tumor volume but without statistically significant values. EOR was the only factor impacting PFS both on univariate (*p* = 0.0002) and multivariate analyses (*p* = 0.0352 HR 1.4554). Details concerning prognostic factors influencing OS are shown in Table 4 and Figure 4.

### 3.3. Postoperative Assessment and Neuropsychological Evaluation

No mortality or major peri-operative morbidity occurred. Postoperative new neurological deficits were observed in six (6.5%) patients (in two cases recovered during RT treatment): motor deficit in two, hemianopsia in two, aphasia in one, and motor deficit plus hemianopsia in one patient. Neuropsychological scores before and after HFRT remained unchanged. The analysis showed no detrimental effect of HFRT on cognitive functions (language, short and long term verbal and visuo-spatial memory, working memory, attentive and executive functions).

## 4. Discussion

The infiltrative behavior of GBM carries a high risk of recurrence, resulting in a short life expectancy [23]. Among the prognostic factors investigated, the extent of surgical resection has proven to influence survival in newly diagnosed GBM patients [23,24,25]. Published data regarding the value of EOR are based on the amount of mass removal only of the contrast-enhancing component. Different authors supposed that FLAIR altered areas could express non-enhancing normal brain pathological invasion and eventually sites of recurrences [26,27,28]. A way forward in improving outcomes might be a more precise identification of the volume at the most significant risk of relapse. The precise definition of the amount of surgical resection, and maybe even more of the residual tumor volume (RTV), is a crucial issue, though, to date, it is a matter of debate. Emerging molecular imaging techniques offer the potential to assess the metabolic tumor status. Positron emission tomography imaging with [11C]-methionine has shown promise for delineating tumor margins, localizing residual tumor sites, and differentiating the residual tumor from reactive changes [29,30]. While MRI gives a morphological tumor delineation, [11C]MET PET provides complementary metabolic information, allowing more precise viable tumor cell identification [6,7]. Evidence regarding the predictive and prognostic value of [11C]MET PET is still lacking, and indications to plan treatments are unclear. In addition, few data regarding the relationship between contrast-enhanced MRI alterations, FLAIR abnormalities and PET uptake are recorded and available regarding newly diagnosed GBMs [8,15,16]. Based on this background, we investigated whether the employment of [11C]MET PET and MRI could provide additional information influencing surgical and RT strategies. Newly diagnosed GBMs patients enrolled in the phase II clinical study received a different amount of surgical resection followed by HFRT with concomitant and adjuvant temozolomide chemotherapy. Considering that the primary aim was to evaluate the benefit of a short course of RT, the results obtained were highly satisfactory with a low rate of side effects [17]. Indeed, although an extensive surgical resection was performed in the largest number of patients treated, no major perioperative morbidity occurred, and adjuvant treatments were started without delays. In addition, notwithstanding, a higher dose on large tumor volume was delivered, including [11C]MET PET uptake, all patients completed the HFRT treatment, no neurological deterioration was observed, and neurocognitive functions remained stable, or in some cases improved. All patients underwent [11C]MET PET for HFRT tumor delineation. A modification of the target volume was recorded in about 11% of patients for [11C]MET PET uptake for up to 30 mm from resections margins included in the FLAIR abnormalities. In this context, further analysis was performed focused on the relationship between the different extents of surgical resection, presence of CERTV on postoperative MRI and [11C]MET PET uptake in terms of biological tumor volume (BTV) and SUVmax. This evaluation aims to define the higher risk areas better and improve surgical and RT planning. As stated by the literature data, the EOR was confirmed as strongly influencing survival when major resections were obtained. Indeed, the median OS time, and the 1 and 2 years OS were 20 months, 73% and 33% for GTR/STR, 13 months, 62.5% and 18.8% for PR and 7 months, 21% and 0 for B (*p* < 0.0001). The presence of CERTV, also in cases of GTR, significantly affected survival with a 3 year OS of 6%, compared to 22% for patients without CERTV. Concerning the use of [11C]MET PET, the following points have to be raised. Firstly, the absence of both RTV and BTV has proven to be a prognostic factor impacting outcomes, with 60% of patients alive at 2 years. Notwithstanding the GTR performed and the absence of CERTV on MRI, 16% of patients had BTV located in FLAIR abnormalities beyond the surgical cavity. In this group, the outcome was worse, compared to the absence of CERTV and BTV, with a 2 year OS of 33% and 60%, respectively. These data underline the importance of metabolic imaging and their influence on patients’ outcomes, suggesting that the FLAIR abnormalities should be, when feasible, included in the surgical resection planning. Considering the risk of morbidity related to the complete resection of FLAIR abnormalities, above all for lesions located near eloquent areas, the MET PET uptake could select the site at greater risk of recurrence and eventually guide neurosurgeons. It is clear that to plan more aggressive resection results, it is pivotal to employ advanced brain mapping. These techniques, eventually to be applied to awake procedures with neuropsychological attendance, if needed, could provide maximal resection with maintenance of full patient functional integrity. Secondly, little data are available regarding the relationship between SUVmax and outcomes. In our analysis, we found that a SUVmax of 5 significantly influenced survival, both on univariate and multivariate analyses (*p* = 0.0059; *p* = 0.0181). However, several studies identified SUVmax limitations, and the calculation of TBR with reproducible methods should be preferred [18,19,31]. Our data showed that the higher the TBRmax, the worse the patients’ outcomes. Specifically, patients with a TBRmax higher than 4.5 showed a significantly worse prognosis than those with TBRmax ≤ 4.5, with a negative trend from 1, 2 and 3 year survival (57.1(±9.3) vs. 70.6(±7.8) months, 10.7(±5.8) vs. 32.4(±8.0) months, 3.5 (±3.5) vs. 14.7(±4.0) months, respectively).

Finally, [11C]MET PET was performed during the follow-up time at 6 and 12 months, or before in doubtful cases of disease progression. Relapse was recorded in BTV in a significant number of cases treated with correspondence between MRI and [11C]MET PET. We are aware that our study’s limits are the low number of patients included, the lack of MET PET in large parts of patients before surgery, the comparison between postoperative MRI performed within 72 h from surgery and [11C]MET PET 1 month after, and the possibility of false positive METPET uptake due to coexistent inflammatory processes. Notwithstanding, our analysis showed that the presence of relivable [11C]MET PET uptake, even in cases of CERTV = 0, was proven to be an unfavorable factor influencing outcome. Our data suggest that 1[11C]MET PET could be an adequate imaging method for tumor volume definition compared to T1-MRI sequences, only. This greater accuracy warrants a reduction in the applied margins and a consequent normal brain tissue sparing for RT planning. Regarding EOR, the use of [11C]MET PET uptake could help identify the area at higher risk of recurrence located in the FLAIR abnormalities, confirming the need to perform surgical resection beyond the enhanced boundaries where uptake is visible. We believe that a correct target volume definition requires different diagnostic complementary modalities, which should be integrated with the clinical evaluation in a multidisciplinary approach.

## 5. Conclusions

In our experience, the use of [11C]MET PET and contrast-enhanced T1 and FLAIR MRI proved to be effective in tumor volume definition for RT planning. This metabolic imaging allowed to detect areas at higher risk of recurrence located in the FLAIR abnormalities, confirming the need to perform surgical resection beyond the enhanced boundaries where uptake is visible. A challenging issue is represented by integrating morphological and functional imaging to better define the actual extent of the surgical resection to perform.

## Figures and Tables

**Table 1:** Patient, tumor and treatment characteristics.

	*n*	%
Patients	93	100
Gender		
Female	33	35.5
Male	60	64.5
Median age (range years)	61 (23–77)	
KPS		
70	6	6.4
80	20	21.6
90–100	67	72
Histology		
Glioblastoma	93	100
Tumor molecular profile		
IDH wild type	93	100
MGMT methylated	53	57
MGMT unmethylated	40	43
Treatments		
Surgical resection	93	100
HFRT	93	100
Total doses/dose per fraction Gy		
CTV1/PTV1	60/4	100
CTV2/PTV2	42/2.8	100
Number of fractions	15	100
Median duration weeks (range weeks)	3 (2.6–4.1)	
Chemotheraphy		
Concomitant temozolomide	91	97.8
Never started concomitant temozolomide	2	2.2
Adjuvant temozolomide	91	97.8
Median number of cycles (range)	6 (1–12)	
Never started adjuvant temozolomide	2	2.2

KPS—Karnofsky performance scale; IDH1—Isocitrate dehydrogenase; MGMT—O-6 methylguanine-DNA methyltransferase; HFRT—hypofractionated radiation therapy; CTV—clinical target volume.

**Table 2:** Extent of resection (EOR), contrast-enhanced residual tumor volume (CERTV) on postoperative MRI and on simulation [11C]METPET characteristics for the whole cohort.

	*n*	%
Patients	93	100
EOR		
GTR	45	48.3
STR	18	19.4
PR	16	17.2
B	14	15.1
CERTV on postoperative MRI		
Yes	63	67.7
No	30	32.3
Median CE RTV on postoperative MRI	4.23 (0.17–35.33)	
BTV on [11C]METPET		
Yes	78	83.9
No	15	16.1
Median BTV on [11C]MET PET	8.47 (0.11–62.20)	
Median SUVmax [11C]MET PET	4.03 (2.30–13.90)	
Median TBRmax	4.34 (2.36–12.00)	
Location of BTV	78	100
CE + FLAIR abnormalities	63	80.8
Only FLAIR abnormalities	15	19.2

EOR—extent of resection; GTR—gross total resection 95–100%; STR—subtotal resection 78–94%; PR—partial resection 30–77%; B—biopsy < 30%; CE RTV—contrast enhanced residual tumor volume; MRI—magnetic resonance imaging; BTV—biological tumor volume; [11C]METPET—11 carbonione methionine-positron emission tomography; TBRmax—maximum tumor-to-background ratio; SUVmax—maximum standardized uptake value.

**Table 3:** Extent of resection (EOR), contrast enhanced residual tumor volume (CERTV), biological tumor volume (BTV), and SUVmax correlations.

	GTR	STR	PR
No patients	45	18	16
Residual Tumor NO (CERTV/BTV)	15	0	0
Residual Tumor YES CERTV + BTV	15	18	16
BTV only	15	0	0
Median SUVmax (range)	3.70 (2.20–9.54)	3.25 (2.3–7.5)	4.7 (2.96–9.30)
Median PFS months (range)	10 (6–22)	11 (2–28)	10 (2–35)

GTR—gross total resection: 95–100%; STR—subtotal resection: 78–95%; PR—partial resection: 30–77%; CERTV—contrast enhanced residual tumor volume; BTV—biological tumor volume.

**Table 4:** Kaplan–Meyer overall survival (OS) according to subgroup analyses.

	Pts	Median OS Months (Months 95% CI)	1 Year OS % (SE)	2 Year OS % (SE)	3 Year OS % (SE)	*p* Value Univariate	HR Multivariate (95% CI)	*p* Value Multivariate
Overall survival	93	16 (14–19)	63.4 (±4.9)	25.8 (±4.5)	10.8 (±3.2)			
Age ≤60 >60	44 49	19 (16–24) 13 (10–16)	77.3 (±6.3) 51.0 (±7.1)	34.1 (±6.4) 18.4 (±5.5)	15.9 (±5.5) 6.1 (±3.4)	**0.0050**	2.6038 (1.5578–4.3524)	**0.0003**
EOR GTR STR PR B	46 17 16 14	21 (18–25) 14 (9–24) 13 (12–18) 7 (6–12)	76.1 (±6.2) 64.7 (±11.6) 62.5 (±12.1) 21.4 (±11.0)	37.0 (±7.1) 23.5 (±10.3) 18.8 (±9.7) 0	15.2 (±5.3) 5.8 (±5.7) 6.2 (±6.0) 0	**<0.0001**	160.2776 (20.3294–1263.6345)	**<0.0001**
MGMT Methylated Unmethylated	53 40	18 (14–23) 15 (12–18)	66.0 (±6.5) 60.0 (±7.7)	34.0 (±6.5) 15.0 (±5.6)	15.1 (±4.9) 5.0 (±3.4)	0.14	0.4626 (0.2776–0.7711)	**0.0031**
CERTV absent present	30 63	23 (19–27) 13 (11-16)	85.2 (±6.5) 54.5 (±6.1)	44.4 (±9.5) 18.2 (±4.7)	22.2 (±8.0) 6.0 (±2.9)	**0.0040**	4.1696 (2.3985–7.2485)	**<0.0001**
BTV absent present	15 78	25 (20–34) 14 (12–18)	100 56.4 (±5.6)	60.0 (±12.6) 19.2 (±4.4)	26.7 (±11.4) 7.6 (±3.0)	**0.0020**	0.4800 (0.3772–0.6109)	**<0.0001**
CERTV/BTV CERTV absent BTV absent CERTV absent BTV present CERTV present BTV present	15 15 63	25 (20–34) 21 (15–26) 13 (11–16)	100 73.3 (±11.4) 52.4 (±6.2)	60.0 (±12.6) 33.3 (±12.2) 15.9 (±4.6)	26.7 (±11.4) 13.3 (±8.7) 6.3 (±3.0)	**0.0029**	1.0728 (0.6131–1.8770)	0.856
SUVmax * >5 2.2–5	18 46	13 (12–16) 19 (12–24)	66.7 (±11.1) 63.0 (±7.1)	5.5 (±5.4) 30.4 (±6.7)	0 13.0 (±4.9)	**0.0059**	2.1071 (1.135–3.909)	**0.0181**
TBRmax * ≤4.5 >4.5	36 28	21 (13–24) 13 (10–16)	70.6(±7.8) 57.1(±9.3)	32.4(±8.0) 10.7(±5.8)	14.7(±4.0) 3.5 (±3.5)	**0.0077**	2.0273 (1.1419–3.5991)	**0.0158**

OS—overall survival; SE—standard error; EOR—extent of resection; GTR—gross total resection: 95–100%; STR—subtotal resection: 78–95%; PR—partial resection: 30–77%; Biopsy: <30%; MGMT—O-6-methylguanine-DNAmethyltransferase; nr—not reached; CERTV—contrast-enhanced residual tumor volume; BTV—residual biological tumor volume; TBR—tumor-to-background ratio; * except biopsy and BTV = 0; bolded numbers—highlight the statistical significance.

## Data Availability

Data supporting reported results can be found in IRCCS Humanitas Research Hospital dataset.

## References

[1] Stupp R., Mason W.P., van den Bent M.J., Weller M., Fisher B., Taphoorn M.J., Belanger K., Brandes A.A., Marosi C., Bogdahn U. (2005). Radiotherapy plus concomitant and adjuvant temozolomide for glioblastoma. N. Engl. J. Med..

[2] Stupp R., Hegi M.E., Mason W.P., van den Bent M.J., Taphoorn M.J., Janzer R.C., Ludwin S.K., Allgeier A., Fisher B., Belanger K. (2009). Effects of radiotherapy with concomitant and adjuvant temozolomide versus radiotherapy alone on survival in glioblastoma in a randomised phase III study: 5-year analysis of the EORTC-NCIC trial. Lancet Oncol..

[3] Stall B., Zach L., Ning H., Ondos J., Arora B., Shankavaram U., Miller R.W., Citrin D., Camphausen K. (2010). Comparison of T2 and FLAIR imaging for target delineation in high grade gliomas. Radiat. Oncol..

[4] De Coene B., Hajnal J.V., Gatehouse P., Longmore D.B., White S.J., Oatridge A., Pennock J.M., Young I.R., Bydde G.M. (1992). MR of the brain using fluid attenuated inversion recovery (FLAIR) pulse sequences. Am. J. Neuroradiol..

[5] NCCN Guidelines Version 1.2021.

[6] Wong T.Z., van der Westhuizen G.J., Coleman R.E. (2002). Positron emission tomography imaging of brain tumors. Neuroimaging Clin. N. Am..

[7] Kubota K. (2001). From tumor biology to clinical Pet: A review of positron emission tomography (PET) in oncology. Ann. Nucl. Med..

[8] Navarria P., Reggiori G., Pessina F., Ascolese A., Tomatis S., Mancosu P., Lobefalo F., Clerici E., Lopci E., Bizzi A. (2014). Investigation on the role of integrated PET/MRI for target volume definition and radiotherapy planning in patients with high grade glioma. Radiother. Oncol..

[9] Riva M., Lopci E., Castellano A., Olivari L., Gallucci M., Pessina F., Fernandes B., Simonelli M., Navarria P., Grimaldi M. (2019). Lower Grade Gliomas: Relationships Between Metabolic and Structural Imaging with Grading and Molecular Factors. World Neurosurg..

[10] Pala A., Reske S.N., Eberhardt N., Scheuerle A., König R., Bernd Schmitz B., Beer A.J., Wirtz C.R., Coburger J. (2019). Diagnostic accuracy of intraoperative perfusion-weighted MRI and 5-aminolevulinic acid in relation to contrast-enhanced intraoperative MRI and 11C methionine positron emission tomography in resection of glioblastoma: A prospective study. Neurosurg. Rev..

[11] Wang Y., Rapalino O., Heidari P., Loeffler J., Shih H.A., Oh K., Mahmood U. (2018). C11 Methionine PET (MET-PET) Imaging of Glioblastoma for Detecting Postoperative Residual Disease and Response to Chemoradiation Therapy. Int. J. Radiat. Oncol. Biol. Phys..

[12] Lundemann M., Cardoso Costa J., Law I., Engelholm S.A., Muhic A., Poulsen H.S., Munck af Rosenschold P. (2017). Patterns of failure for patients with glioblastoma following O-(2-[18F] fluoroethyl)-L-tyrosine PET- and MRI-guided radiotherapy. Radiother Oncol..

[13] Lee I.H., Piert M., Gomez-Hassan D., Junck L., Rogers L., Hayman J., Haken R.K.T., Lawrence T.S., Cao Y., Tsien C. (2009). Association of 11c-methionine pet uptake with site of failure after Concurrent temozolomide and radiation for primary glioblastoma multiforme. Int. J. Radiat. Oncol. Biol. Phys..

[14] Iuchi T., Hatano K., Uchino Y., Itami M., Hasegawa Y., Kawasaki K., Sakaida T., Hara R. (2015). Methionine Uptake and Required Radiation Dose to Control Glioblastoma. Int. J. Radiat. Oncol. Biol. Phys..

[15] Harat M., Małkowski B., Makarewicz R. (2016). Pre-irradiation tumour volumes defined by MRI and dual time-point FET-PET for the prediction of glioblastoma multiforme recurrence: A prospective study. Radiother. Oncol..

[16] Grosu A.L., Weber W.A., Riedel E., Jeremic B., Nieder C., Franz M., Gumprecht H., Jaeger R., Schwaiger M., Molls M. (2005). L-(methyl-11C) methionine positron emission tomography for target delineation in resected high-grade gliomas before radiotherapy. Int. J. Radiat. Oncol. Biol. Phys..

[17] Navarria P., Pessina F., Tomatis S., Soffietti R., Grimaldi M., Lopci E., Chiti A., Leonetti A., Casarotti A., Rossi M. (2017). Are three weeks hypofractionated radiation therapy (HFRT) comparable to six weeks for newly diagnosed glioblastoma patients? Results of a phase II study. Oncotarget.

[18] Unterrainer M., Vettermann F., Brendel M., Holzgreve A., Lifschitz M., Zähringer M., Suchorska B., Wenter V., Illigens B.M., Bartenstein P. (2017). Towards standardization of ^18^ F-FET PET imaging: Do we need a consistent method of background activity assessment?. EJNMMI Res..

[19] Law I., Albert N.L., Arbizu J., Boellaard R., Drzezga A., Galldiks N., la Fougère C., Langen K.J., Lopci E., Lowe V. (2019). Joint EANM/EANO/RANO practice guidelines/SNMMI procedure standards for imaging of gliomas using PET with radiolabelled amino acids and [^18^ F]FDG: Version 1.0. Eur. J. Nucl. Med. Mol. Imaging.

[20] Wen P.Y., Macdonald D.R., Reardon D.A., Cloughesy T., Sorensen A., Galanis E., Degroot J., Wick W., Gilbert M., Lassman A. (2010). Updated response assessment criteria for high-grade gliomas: Response assessment in neuro-oncology working group. J. Clin. Oncol..

[21] Papagno C., Casarotti A., Comi A., Gallucci M., Riva M., Bello L. (2012). Measuring clinical outcomes in neuro-oncology. A battery of evaluate low-grade glioma (LGG). J. Neurooncol..

[22] Cox D.R. (1972). Regression models and life tables. J. R. Stat. Soc..

[23] Lacroix M., Abi-Said D., Fourney D.R., Gokaslan Z.L., Shi W., DeMonte F., Lang F.F., McCutcheon I.E., Hassenbusch S.J., Holland E. (2001). 2001 A multivariate analysis of 416 patients with glioblastoma multiforme: Prognosis, extent of resection, and survival. J. Neurosurg..

[24] Chaichana K.L., Jusue-Torres I., Navarro-Ramirez R., Raza S.M., Pascual-Gallego M., Ibrahim A., Hernandez-Hermann M., Gomez L., Ye X., Weingart J.D. (2014). Establishing percent resection and residual volume thresholds affecting survival and recurrence for patients with newly diagnosed intracranial glioblastoma. Neuro Oncol..

[25] Pessina F., Navarria P., Cozzi L., Ascolese A., Simonelli M., Santoro A., Clerici E., Rossi M., Scorsetti M., Bello L. (2017). Maximize surgical resection beyond contrast-enhancing boundaries in newly diagnosed glioblastoma multiforme: Is it useful and safe? A single institution retrospective experience. J. Neurooncol..

[26] Li Y.M., Suki D., Hess K., Sawaya R. (2016). The influence of maximum safe resection of glioblastoma on survival in 1229 patients: Can we do better than gross-total resection?. J. Neurosurg..

[27] Duffau H. (2014). Is supratotal resection of glioblastoma in noneloquent areas possible?. World Neurosurg..

[28] Wilson T.A., Karajannis M.A., Harter D.H. (2014). Glioblastoma multiforme: State of the art and future therapeutics. Surg. Neurol. Int..

[29] Kato T., Shinoda J., Nakayama N., Miwa K., Okumura A., Yano H., Yoshimura S., Maruyama T., Muragaki Y., Iwama T. (2008). Metabolic assessment of gliomas using 11C-methionine, [18F] fluorodeoxyglucose, and 11C-choline positron-emission tomography. Am. J. Neuroradiol..

[30] Kracht L.W., Miletic H., Busch S., Jacobs A.H., Voges J., Hoevels M., Klein J.C., Herholz K., Heiss W.D. (2004). Delineation of brain tumor extent with [11C]L-methionine positron emission tomography: Local comparison with stereotactic histopathology. Clin. Cancer Res..

[31] Ferjančič P., Ebert M.A., Francis R., Nowak A.K., Jeraj R. (2021). Repeatability of Quantitative 18F-FET PET in Glioblastoma. Biomed. Phys. Eng. Express..

